# "The Hospital" Nursing Mirror

**Published:** 1897-03-13

**Authors:** 


"The Hospital" Nursing Mirror.

Being the Nursing Section of "The Hospital."

[Contributions for this Section of "The Hospital" should be addressed to the Editor, The Hospital, 28 & 29, Southampton Street, Strand, London, W.C., and should have the word "Nursing" plainly written in left-hand top corner of the envelope.]

### News from the Nursing World.

### THE QUEEN'S COMMEMORATION IN THE COLONIES.

The desire to commemorate the Queen's long reign by the establishment of nursing institutions is not confined to the British Isles, but is extending to her colonies and dependencies. In Montreal, for instance, it has been proposed to build a nurses' home in connection with the General Hospital; and Lady Aberdeen is organising a scheme of district nursing in Canada.

### THE LONDON COUNTY COUNCIL AND ASYLUM ATTENDANTS.

Mr. Crooks' long-deferred motion in favour of reduced hours for attendants in asylums under the London County Council came up for consideration at a recent meeting. A fourteen hours' working day is an admitted fact in the case of a considerable number of these unlucky workers; and Mr. Crooks instanced the admirable working of King's College Hospital, where the nurses have what is practically an eight hours' day, as a proof of the feasibility of his proposals. Nevertheless, the prospect of the increased staff which a change would necessitate, and the consequent cost, frightened the economical mind of the Council. Mr. Crooks' proposals fell through, and the London County Council will continue to sweat its workers for the present.

### DISTRICT NURSES' HOME FOR WESTMINSTER.

A public meeting of Westminster parishioners was held on March 5th to "consider what steps shall be taken to commemorate the completion of the sixtieth year of the Queen's illustrious and beneficent reign." Mr. Burdett-Coutts presided. The following resolution was unanimously adopted, moved by Canon Wilberforce, and seconded by Mr. Wolfe Barry: "That the parishioners be invited to subscribe to a fund for erecting, or otherwise providing, a freehold house, in a central part of the parish, as a home for trained nurses for the sick poor at their own homes, and for the perpetual engagement of a trained nurse in addition to those now acting under the direction of the Westminster Nursing Committee, such home and nurse to be designated by a title fittingly identifying them respectively with the happy occasion."

### THE SICK CHILDREN'S ASYLUM BOARD FOR LONDON.

The State Children's Aid Association has issued a leaflet, which sets forth very clearly the advantages and disadvantages of the proposed Sick Children's Asylum Board for London, which all who are interested in this most important problem of how to deal wisely and well with 17,000 pauper children in barrack schools, workhouses, and infirmaries, should not fail to study. The leaflet concludes with these words: "As the disadvantages of the proposed Sick Children's Asylum Board outnumber and outweigh the advantages, all interested in the welfare of the State-supported children are advised to use such influence as they possess to oppose this 'draft order' becoming a permanent one, and that every effort should be made to induce the Government to create a strong, responsible central metropolitan board, endowed with possession of the buildings, appliances, and staff, and thus enabled to classify the children, to sell some of the buildings, and to utilise others for the varied purposes for which provision is required to meet the changing needs of a child's life."

### THE VICTORIA HOSPITAL FETE.

The Imperial Fête and Fancy Fair in aid of the Victoria Hospital is going to be a wonderful show, and taking place in just the week of all the year when London will be most filled to overflowing with comers from all parts of the world, it should prove all its energetic organiser, Commander Blount, can wish. Nurses will be interested to note this item in the long programme of entertainments: "Readings—'Stories from a Children's Hospital'; dedicated by special permission to H.R.H. Princess Louise, Marchioness of Lorne, by Miss Gethen, formerly nurse at the Victoria Hospital, and Sister Queen Victoria at the London Hospital."

### NURSING AT GUY'S HOSPITAL.

The nursing staff at Guy's Hospital is being augmented by additional night nurses, and a second night sister has been appointed. One sister will now have the superintendence of the medical building and clinical ward, and the other that of the surgical building.

### MIDWIFERY TRAINING.

Many are the questions we receive from correspondents anent midwifery training, especially as to where "free training" may be obtained, or at least "the very cheapest." We have often stated that there is *no* free training to be had, but the stream of questions on this point still flows. In *Nursing Notes* (the journal of the Midwives Institute) for March a comment appears on the same subject, advising those who wish for training in hospital to write to the four chief lying-in hospitals for their terms. For those who wish to train in district midwifery, various directions may be obtained from the Institute, 12, Buckingham Street, and they are informed that the expense of the training varies from 15 to 18 guineas.

### QUEEN'S NURSES AT STAMFORD.

The committee of the Stamford Branch of Queen Victoria's Jubilee Institute for Nurses have been able to submit a very satisfactory report of last year's work. The balance in hand last year was £5 3s. 3½d., making, with the receipts from all sources, a total income of £106 19s. 2½d. The expenditure amounted to £91 16s. 10d., so that the beginning of the present year found the society with a balance in hand of £15 2s. 4d. The Queen's inspector reported Nurse Blackmore's work to be "very good," and her services most "acceptable to the people." Nurse Blackmore was amongst the nurses invited to Windsor last summer.

### A NOBLE VOLUNTEER.

Many "Old Londoners" will be interested to hear that Miss Catherine McIntosh, formerly "Nurse Coope" at the London Hospital, and since 1890 Sister at the Government Civil Hospital, Hong Kong, sailed last week for Bombay, having volunteered her services to Government to help in nursing the victims of the plague. Miss McIntosh was at her post at Hong Kong throughout the terrible epidemic there in 1895, had the disease herself, and was sent home on furlough and sick leave. Six months of her leave only have elapsed, but being now quite well, with that devotion to duty and self-forgetfulness which nurses are ever ready to show in times of need, Miss McIntosh thought it right to offer to go to India if nurses were really needed. She wrote direct to Mr. Chamberlain (as Colonial Secretary), who gave permission for her temporary transfer to the Indian Government, and the whole matter was settled with great promptitude. Of course, her experience will be of the utmost value, and the Indian authorities have done wisely to secure her services.

### DISTRICT NURSING AT SWANSEA.

Dr. Jabez Thomas, a member of the medical staff of the Swansea General Hospital, has suggested a scheme for the provision of fully-trained nurses for the rich and the poor of Swansea which deserves consideration. It has been proposed to celebrate the present year by raising a fund to place the hospital "on a sound financial basis," and Dr. Thomas adds to this the idea of establishing a nurses' home and institute in connection with the hospital. This scheme he would wish to see started on the lines of the Guy's Hospital Institute for Nurses, providing, besides private nurses, district nurses to follow the hospital cases to their own homes when necessary, and to be carried out on a scale which should ensure a good supply of the best nursing for the whole town.

### MEDICO-PSYCHOLOGICAL ASSOCIATION.

The next examination for the Medico-Psychological Association's certificate in Nursing and Attending on the Insane will be held on Monday, May 3rd. Schedules to be filled in by intending candidates can be obtained from Dr. Spence, Burntwood Asylum, Lichfield. These must be returned four weeks before the examination, Monday, April 5th, being the last day upon which candidates can enter their names for the May examination.

### THE VICTORIAN ERA EXHIBITION.

The Nursing Section of the forthcoming Victorian Era Exhibition is sure to be extremely interesting, and is being well worked up. The fact that trained nursing is so eminently a product of the "Victorian Era" makes it right and fitting that the section devoted to it shall be well to the fore at Earl's Court this summer. Amongst the exhibits there will be, of course, many connected with Miss Nightingale, notably a bust given to her by the soldiers who fought in the Crimea, and some early portraits.

### PRIVATE NURSING IN PARIS.

A correspondent inquired the other day if there were any openings in Paris for English private nurses, or for "nursing homes." A nurse who has worked in that city for several years sends us some information on the subject. There are (she says) a great many English private nurses in Paris, really more than are needed, and newcomers must be prepared to wait sometimes a weary while before they get cases, the doctors being very loyal to those who have worked with them. Besides the Hollond Institute, there are two or three "settlements," as it were, of English nurses—friends who live together, and add to their number as seems advisable. The last year has been a hard one for many nurses in Paris, some who are trying to make a start in private work being for months without a case. Nurses to be successful must be very adaptable people, ready to fall in with the ways of French people and to work for French doctors without giving way to the common English failing of believing English ways, and English ways only, to be the right ones. For private institutions for paying patients there would seem to be little scope. The Hertford Hospital takes in well-to-do patients, and most of the great surgeons have *cliniques* of their own, where they prefer to have their private patients. There are already several homes for the especial benefit of English and American people. Altogether, it would appear that English nurses are not at present in demand in Paris, and that only those with very special qualifications and a certainty of a good opening should dream of making such a venture.

### SHORT ITEMS.

A concert was given in aid of the Gainsborough Nursing Association on March 1st.—A very telling little duologue, with Mr. Hall Caine and Nurses for its subject, appeared in a recent number of *To-Day*.—The Richmond Board of Guardians have decided to make a grant of £10 to the Mortlake Nursing Association.—It has been announced that Lord Iveagh has given £12,500 to "the Commemoration Fund for providing trained Queen's nurses for the Irish poor."—A successful performance of "The Yeomen of the Guard" was given by a number of ladies and gentlemen the other day in aid of the Hampstead "Nursing Appliances Fund." During the past year the Fund has been the means of helping 99 cases from Kentish Town and the neighbourhood of the Victoria Docks, while 26 patients have been sent to a convalescent home at Herne Bay, where a bed is maintained by the fund.—At a recent meeting at Brighton it was resolved to devote any money subscribed over and above the amount required for the festivities in celebration of the Queen's reign to the fund for the Nurses' Home at the Sussex County Hospital. More than £1,000 has been already received. —A very successful concert was given by Mr. Percy Armytage's "Children's Orchestra" on March 6th, in aid of the Prince of Wales' Hospital Fund. The concert was held at 1, Belgrave Square, by permission of Mrs. Sassoon.—Miss Tanner, Lady Superintendent of the Institution of Trained Nurses, Aylestone Road, Leicester, has resigned her appointment. She was presented, on leaving, with a silver card-case by past and present members of the staff "as a parting gift of love and gratitude."—The Clothworkers' Company have sent a donation of £250 in aid of the Extension and Improvement Fund of Queen Charlotte's Lying-in Hospital, Marylebone Road.—Her Royal Highness Princess Christian of Schleswig-Holstein has kindly consented to become a patron of Friedenheim, the Home of Peace for the Dying, Swiss Cottage.—We have just received the secretary's report of the fourth annual Convention of Superintendents of American Training Schools for Nurses, held at Baltimore on February 10th. Nearly fifty members were present, and several visitors. On the 11th and 12th a second meeting was held of the convention instituted a year ago by this society for the purpose of organising an association of graduate nurses in America.

### Pharmacy and Dispensing for Nurses.

By C. J. S. Thompson.

### V. — SIALAGOGUES — STIMULANTS — STYPTICS — TONICS—GUIDE FOR STUDY AND QUALIFICATIONS.

Sialagogues are medicines that increase the secretion of the saliva. They include ether, horseradish, mercury and its salts, the iodides, jaborandi, mezereum, calabar bean, pilocarpine, pepper, pellitory, rhubarb, mustard, tobacco, and ginger.

Stimulants increase the natural function of a part, or which cause a slight amount of superficial irritation. They are classed as follows: General Stimulants — Alcohol in small doses, ether, ammonia, arnica, cajepute oil, and phosphorus. Spinal Stimulants — Benzoic acid, ether, arnica, ammonium carbonate, belladonna, Indian hemp, cantharides, ergot, morphine, nux vomica, cajepute oil, opium, phosphorus, strychnine. Vascular Stimulants — Alcohol, ether, ammonia, castoreum, galbanum, guaiacum, mezereum, sassafras, sumbul, oil of turpentine, and valerian.

Styptics are medicinal agents which arrest bleeding. They include gallic acid (valuable remedy for internal hæmorrhage), tannic acid, alum, and matico.

Tonics are medicines which impart tone or strength to the body or its parts. They may be classed under the following heads: Tonics that act through the blood and improve its quality—Preparations of iron, cod liver oil, and arsenic. Nervine Tonics—Arsenic, nitrate of silver, oxide of silver, oxalate of cerium, cinchona bark and its preparations, coca, copper sulphate, damiana, iron salts, nux vomica, phosphorus, sodium and calcium hypophosphites, strychnine, zinc acetate, oxide, and sulphate. Stomachic and Intestinal Tonics—Acids: Hydrochloric diluted, nitric diluted, nitro-hydrochloric diluted, phosphoric diluted, sulphuric diluted, chamomile, orange peel, balsam of Peru, beberine sulphate, buchu, calumba, canella, cascarilla, chiretta, cinchona bark, cinchonidine, cinchonine, cusparia, compound decoction of aloes, gentian, guarana, hydrastin, lemon peel, hop, menyanthes, winter's bark, nux vomica, pepsine, peptonised foods, pareira, quassia, quinine sulphate, rhubarb, salicin, scopentaria, and strychnine. Vascular Tonics—Adonis, apocynum, caffeine, convallaria, digitalis, iron salts, nux vomica, squill, sparteina, strophanthus, strychnine, and green hellebore.

Having enumerated most of the drugs and preparations employed in the practice of medicine, with which it is necessary for the student to become familiar, it will be well, before proceeding to deal with the practical art of dispensing, to indicate a guide to the study of the several subjects bearing on pharmacy, of which a knowledge is necessary.

To commence with, an intimate knowledge of the British Pharmacopœia and its preparations, with their doses, is most essential. A good method of impressing these on the memory is to write down the English and Latin names of the drugs and chemicals, followed by the preparations they enter into, and their doses. To gain an insight into pharmaceutical and practical chemistry, Attfield's work on this subject will be found most useful. The processes for manufacturing all the chemicals included in the pharmacopœia, together with the general laws relating to chemistry and pharmacy, are given in this treatise.

Materia medica must also receive attention, especially that of the organic or vegetable kingdom, and some knowledge of the active principles of drugs and the methods of judging their quality and freedom from adulteration acquired. For this purpose Maisch's or Garrod's Materia Medica are recommended. For the study of botany the student cannot do better than use "Scott's Structural Botany" or "Green's Manual," aided by practical examinations of plants and specimens. "Pareira's Selecta è Prescriptis" is a useful little work, which contains the usual terms employed in prescriptions, and from which instruction in reading and translating Latin prescriptions may be gained. Proctor's "Practical Pharmacy" is also a valuable work on that subject, and the writer's "Practical Dispensing" should be carefully studied.

Besides the systematic study of the subjects mentioned, a course of instruction under a qualified pharmacist is necessary, in order to learn the many technicalities connected with the art, which can only be gained by practical experience.

Should the nurse wish to qualify as a dispenser of medicines in this country, it is necessary to enter for the following examinations, which are held under the direction of the Pharmaceutical Society of Great Britain, which is empowered by the Legislature to hold examinations for those who desire to become registered pharmaceutical chemists, and chemists and druggists or compounders of medical prescriptions. The first or preliminary examination is held four times in the year in most of the larger centres. The examination is wholly in writing, the subjects being Latin, arithmetic, and English. Certificates of having passed the Local Junior or Senior Oxford or Cambridge, or similar examinations in which the above subjects are included, are accepted in lieu of the first examination. The second or minor examination is also held four times during the year in London and Edinburgh only. This examination is divided into two parts, the first being written and practical, and the second oral. The candidate must have attained the age of 21 years, and must produce a certified declaration of having been practically engaged in the translation and dispensing of prescriptions for a period of three years. The subjects comprised are: (1.) Chemistry and physics. (2.) Practical chemistry, including the determination of the specific gravity of liquids and solids, the detection of the chief impurities in the drugs and chemicals included in the British Pharmacopœia, and the candidate must also be able to perform qualitative and volumetric analysis, &c. (3.) Botany, including the recognition of plants and a general knowledge of structural botany and the physiology of plants. This subject includes practical work with the microscope. (4.) Materia medica includes the recognition of crude drugs and the detection of their adulterations. Their geographical sources and botanical and zoological names must also be given, &c. (5.) Prescription reading and translating, and the detection of errors and unusual doses is necessary. The candidate is also examined in practical dispensing. (6.) Pharmacy includes a knowledge of the various operations and processes necessary in manufacturing pharmaceutical preparations and the principles involved in the dispensing of medicines, such as the best excipients and methods for forming pill masses, the preparation and nature of emulsions and the most suitable agents for that purpose, and the best means of suspending insoluble substances liquids. Those who pass this examination are registered and qualified as chemists and druggists, and may practice as such or dispensers of medicine. The third or major examination for registration as a pharmaceutical chemist, demands a more advanced knowledge in chemistry, physics, practical chemistry, botany, and materia medica.

The Society of Apothecaries hold an examination, and grant a certificate to those who pass, as "assistants to apothecaries," but this examination does not qualify for registration under the Pharmacy Act.

### Post-Graduate Clinics for Nurses.

By a Trained Nurse.

### V.—SOME POINTS FOR PRIVATE NURSES.

Again, in the matter of "toileting" a patient in private practice, there is much indeed to be acquired by a nurse fresh from Hospital. And one of the first prejudices the nurse must divest herself of in this connection is a dread which possesses many lest a too great personal care of a patient is *infra dig.* and derogatory. This is a common—and perhaps natural—error, into which many a young practitioner falls, and it is one which, unfortunately, all do not shake off.

Nurses frequently say, "You see it is so different working for the rich and the poor. So long as we are in Hospital we do not mind what we do for the sick, because there charity and philanthropy come in. So that when we do little extra personal things for our patient—dressing off a woman's hair, &c.—we know she does not think we do it because we are inferior to her; she recognises it is done just out of kindness. But when we do the same things for ladies, we are so afraid they will think it is a question of class, and will regard us as a kind of superior lady's maid."

This complaint has been made in my presence perhaps a score of times, and has always appeared to me to be a quite unintelligible fear, arising partly from a want of worldly experience, and partly from a misconception of what is and what is not "professional."

Now it must surely suggest itself as an axiom for universal acceptance that to make her patient happy and content is one of the first items on the programme of a nurse. And so long as she is thinking of this, and using all her best interests and energies in the nursing of her case, she will assuredly be a happy and a dignified professional woman.

When once little hair-splittings about what is derogatory and "degrading" begin to crop up between patient and nurse the dignity and harmony of the sick-room are banished, and a miniature battlefield set up wherein small warring elements raise serious barriers to successful nursing. There are certain defined rules which have rightly been established for the protection of patient and nurse alike, and these must, on occasion, be invoked. But a little tact and kindheartedness on the part of the nurse to her sick patient—and it is a great help when dealing with patients to remember they *are* sick, a little point often overlooked when they appear unreasonable and trying—will generally smooth over difficulties, and render appeals unto Cæsar quite unnecessary.

I have been asked by so many nurses to deal with this question of the personal care of the sick, how far it should go, and where it should be bounded, that I am convinced the question is rather difficult of solution to many. And, indeed, I have met with so many instances of difficulty in this matter that I think it would be interesting to deal with the question from several standpoints.

A few days since I was discussing this very subject with a highly cultivated and admirable American nurse, whose views on the subject would, I knew, be of interest to the readers of The Hospital, although these may not accord with their own. So far as possible her interpretation of how far the trained nurse is bound to give "toilet" assistance to her patients, is reproduced in her own words. She began: "I would like to say, if you sift this matter down—and this I propose to do at the risk of offending the prejudices and fine tastes of some of your readers—that a trained nurse who elects to do private nursing ought to have a little of the instinct of the lady's maid in her composition just in the same way as she should possess the finer feelings of a cook: those feelings which help to guide her patient's tastes in the matter of food, and to see that all he gets is of the finest and best kind. Now, remember I am an American, and as such am accustomed to saying what I think in a perfectly straightforward way. You know in the States we do not wrap things up so much in pink cotton wool and fancy fixings as you do here. If we think a thing we say it. Now, from what I have seen of a good many private nurses in London in their relation to their patients, it seems to me that their training in the personal care of their patients is not so good as with us. For many years I have been a private nurse in New York City, and have always taken great pride in making my women patients look nice by dressing them up in their prettiest and best, not only when convalescing but when they were in bed. And I have taken special pains to put up their hair daintily and becomingly all the time they were under my care. And sometimes, when they have been getting better and I have had more time to spend over their *coiffeur*, they have declared they never looked so well even when their hair was dressed for big balls by leading hairdressers.

Now I yield to no one in my professional pride, and I do not allow that, by making my patient look dainty and nice, I am in any sense putting myself on a level with a lady's maid. I do it because I like my patients, and take a pride in making them look nice. Having been in London two years, with my eyes very wide open, I have had an opportunity of seeing a great many private patients, and, speaking generally, I have been much astonished at the slipshod manner in which many of the English private nurses "dress off" their patients. While in bed the women's hair is either in two ugly plaits, or is "dumped" together in a hideous kind of "bun" at the top of the head! When convalescing the state of things is not much better. The hair is caught up anyhow, long straggles of fringe stray about, and the convalescing woman has a horrible conviction that her illness has destroyed all her good looks, and that she has "gone off" horribly! But her apparent ugliness all arises from the want of a little lady's-maiding. Frequently I have suggested to such private nurses that they should take a lesson in hair dressing, and they have been scornfully indignant. There are many other ways in which some of these nurses show that the "aids to the toilet" of a refined woman are not known to them. And I think it tells against them in their profession. Of course, we know that a large percentage of the upper classes in England have lady's maids, but there are thousands of women whose husbands are in the professions—women who are cultivated and nice, and who, therefore do not care during their illnesses to be converted into 'frights' through the want of just a little nicety and skill on the part of their trained nurses."

I wish this American nurse could be persuaded to read a paper on this very interesting question. It would at any rate be original and suggestive, though I fear her very decided views would meet with a good deal of opposition. Of course her assertions are too sweeping, as the assertions of very downright people are apt to be. But there is an enormous amount of truth underlying her statements as to the neglect of prettinesses and dainty finishes which many private nurses, though most artistic in feeling and truly kind in disposition, are afraid to give their patients lest it might not be "nurse-like" and professional.

*(To be continued.)*

### A very "Peculiar" Home of Rest.

It clearly behoves nurses and others who are seeking for change of air to exercise a considerable amount of caution in their choice of a "Home of Rest" in which to obtain it, for, from the account of an inquest held in Dover on March 2nd, it would appear that places exist where peculiar views obtain as to the advantage of medical aid in sickness, at any rate for the inmates. In the case in question these views were carried to such an extreme that a nurse who had been invited to enter Mrs. Baker's Home of Rest, 77, St. James' Street, Dover, was allowed to die there from typhoid fever without any medical aid being summoned, although it was admitted that she became delirious before she died. It seems that the people who manage these homes, although not admitting that they are members of the Peculiar People, are afflicted with the same craze, and most people will agree with the sensible verdict brought in by the jury on this poor girl—a nurse at the Mildmay Hospital, London—who went down hoping for health, and was cruelly allowed to drift into death without even such medical attendance as is the right of every pauper in the land. According to the *Dover and County Chronicle*, the jury found that the deceased died from typhoid fever, and added a rider to the effect that it was consequent of the neglect on the part of the authorities of the home in not calling in medical aid. Little note seems to have been taken of the fact that the case was not notified to the sanitary authorities, who, however, it is to be hoped, will take action in the matter, for it was admitted in the evidence that the patient was known to be suffering from typhoid fever, and it is extremely important that the duty imposed upon the householder by the Notification Act should be properly enforced.

One of the strangest things in this disgraceful affair is the fact that in the Home of Rest there was an inmate who was stated to be a nurse employed at the North-Western Hospital, and who, according to the evidence of the lady superintendent, "took charge" of the deceased. The conduct of this woman was most extraordinary, and no doubt many nurses will consider it so out of the common as to have in it no lesson for them. What we would wish, however, to impress upon nurses at large is that, although this sad case may seem quite out of the ordinary course, it does convey a lesson to nurses at large in regard to what is a great temptation to some nurses, and a pet weakness of not a few, namely, the tendency to overstep their duties and assume functions which belong to medical practitioners. In this case the nurse apparently "took charge" of the case, and it "did not occur to" her to suggest a doctor. In thus acting she was clearly carrying out the traditions of the home. The superintendent said they "were trusting to the Lord," and the owner of the home said that she "would rather have prayers and the Word of God than a doctor," and that "those who have received the Spirit of God do not need" a doctor. Under such circumstances one might have expected that the nurse, who was so evidently carrying out the wishes and intentions of the "authorities," would have been supported by them, so far as they could give any support under such circumstances, and that the owner who, according to the foreman of the jury, seemed to have the entire management of the home, would have accepted the blame attached to the system on which it was carried on. But not at all! When the real point came the responsibility was thrown on the nurse. Mrs. Baker, to whom the home belongs, seems to have said, "Well, if the trained nurse had not been there it would have been different. I should then have sent for a doctor. The Coroner: Would you have allowed your own child to die in that way? Witness (evasively): I can't say. If the nurse had not been there it would have been quite different." Thus the presence of the nurse was made the excuse for this neglect; and it wil always be so. If nurses overstep their duties they gain for a time a little cheap popularity among the ignorant and the interested, but they may rest assured that, if anything once goes wrong, their friends and patrons who have praised and flattered them as being so "nice and useful," will turn upon them directly, and cast on them all the responsibility for the untoward results of their wrong doing, however good natured may have been the motives which have led them to exceed their duties.

### Nurses for the Plague.

Last week the Indian Government requested the India Office to send six nurses to Bombay. At the beginning of this week we ascertained that three nurses had been selected, and that three others were waiting approval. These, we learn, have now been appointed, and the six sail on Friday in the "Himalaya" for Bombay. Their names are: Miss M. Hale, trained at the West London Hospital; Miss A. Jones, trained at the Royal Free Hospital; Miss J. E. Wheatley, St. George's Hospital; Miss M. C. Steele, St. Bartholomew's Hospital; Miss F. Coleman, St. Bartholomew's Hospital; and Miss L. M. Robinson, Ryde General Hospital.

The engagement is for one year (except in case of misconduct or breakdown of health); free passage by P. and O. steamer to India and back is allowed, with incidental travelling expenses, outfit allowance, and pay of Rs. 175 per month, in addition to free quarters, fuel, lights, &c., to commence from date of embarkation. The Government have also despatched to Bombay another volunteer, Miss Catherine McIntosh, one of the sisters at the Government Civil Hospital at Hong Kong, who had a good deal of experience in plague nursing when the disease was prevalent there. She has volunteered to give up the remaining six months of a year's furlough and go to Bombay on her way back to Hong Kong.

### Where to Go.

The Trained Nurses' Club, 12, Buckingham Street, Strand.—Courses of classes are being held under the auspices of the Society of Trained Masseuses, for "Instruction in the physical exercises and movements required in the treatment of curvature of the spine." The training includes instruction in the general anatomy of the spine and pelvis, and demonstrations on the nature and different forms of spinal curvature, the object being to afford masseuses and intending masseuses an opportunity of qualifying to carry out, under medical direction, this treatment in spinal cases, combined with massage. The fee for the course is four guineas. Examinations are held for certificates, and the capable instructor is Miss Dove, an associate of the Society of Trained Masseuses.

The Victoria Commemoration Club, 29, Southampton Street, Strand.—Miss Thomson is now organising classes of plain and invalid cookery, and will be glad to receive the names of those who may wish to attend either course, with their suggestions as to the most suitable days and hours on which these should be held.—The next lecture at the club will be delivered by Miss Mollony (Society of Trained Masseuses), on Tuesday, March 16th, at five p.m., and will be on "The Training of Masseuses; The Possibilities of Massage, its Limits, &c."—On Wednesday, the 17th, Charles Heath, Esq., F.R.C.S., will deliver a lecture on "The Duties which Nurses may be called upon to Perform in the Nursing of Cases with Disease of the Throat, Nose, or Ear."—Tickets for the lectures may be obtained from the Secretary at the Club, 1s. each to members; 1s. 6d. to non-members. If to be sent by post, a stamped envelope must accompany the application. It will be of great assistance to the secretary if, as far as possible, application for tickets can be made before the date of the lecture.—Miss Earle, of the National Training School of Cookery, gave a most fascinating practical demonstration on the cooking of "Dainty Dishes for Invalids" at this club on Tuesday, March 9th. At the close of the demonstration the audience requested to be allowed to purchase the dishes made.

### The Colonial Nursing Association.

It will be remembered that this association began its public career last summer, inaugurated by a drawing-room meeting in July, over which Lord Loch presided, and at which a number of influential people were present. This was the first public introduction of the association to the outside world, as it were; but before this two nurses, whose salary and maintenance had been kindly guaranteed by one interested in the question of supplying nurses to the colonies, had sailed for Mauritius, and had there been so constantly employed that no need had arisen to call upon the guarantee fund, their earnings, together with the local fund in the colony, proving sufficient to cover all expenses.

In December last the colonies of Cyprus, Sierra Leone, St. Vincent, Dominica, and Newfoundland applied to the association for help, and, preliminary arrangements having been completed, nurses have been engaged and sent out to these places. Cyprus, Sierra Leone, St. Vincent, and Newfoundland have availed themselves of the guarantee fund, for, being very poor colonies, with small shifting communities, without such assistance it would have been impossible for them to achieve trained nursing, urgent though the need is proved to be.

It must be understood that in some colonies no Government funds are available for this purpose, and although the wife of the Governor is organising the movement in each case, it has not been found possible to raise subscriptions in these communities to cover the cost of maintenance and salaries (£60 per annum for each nurse). The C.N.A. has, therefore, to supply the deficit or let the scheme fall through.

In December the Colonial Office addressed the Colonial Nursing Association on the subject of the engagement of four nurses for service in the Government hospitals of Accra (Gold Coast) and Lagos, a new departure on the part of the Gold Coast Government directly owing to the expression of Mr. Chamberlain's wishes on the subject in his circular despatch to the Governors of Crown Colonies last year, at the request of the association. Four suitable candidates were selected by the committee, all of whom were engaged by the Colonial Office. These nurses have now sailed for their various posts. In their cases the Colonies provide the funds, the Colonial Nursing Association merely selecting suitable candidates for appointment, having placed the services of the association at the disposal of the Colonial Office in all matters connected with nursing abroad. The association has now nine nurses working in various Colonies.

The Agent-General for the Cape and Natal are making inquiries with a view to avail themselves of the organisation for the future provision of nurses in South Africa. The Colonial Nursing Association may, in fact, now be considered the recognised organisation for supplying, and, where necessary, assisting financially in the provision of, trained nurses for work in our Colonies.

It is intended to hold a public meeting some time in May or June (of which the details are not yet settled) to make application for more funds, the small amount subscribed more or less privately last year, amounting to about £600, having now too many claims upon it to wait until 1898. The work of the association is sure to commend itself heartily to all those interested on their own or on their friends' account in Greater Britain beyond the seas, and may confidently look for generous support when their work and its needs are more generally known. The association has an enthusiastic and devoted hon. secretary in Mrs. Piggott, to whose exertions the scheme owes its initiation. The address of the association is at the Imperial Institute, S.W.

### Workhouse Infirmary Nursing Association.

The report of this association for 1896, just issued, shows very plainly how great are the difficulties which surround the whole question of workhouse nursing at the present time. Since its establishment in 1879, "in order to supply boards of guardians with competent and duly trained nurses," the association has done wonders, and the fact that boards of guardians, who have learned the value of its nurses, will raise salaries in order to secure them, speaks for itself. The association has done, and is doing, a most important work in training probationers for workhouse nursing, and supplying them to country unions, binding them to Poor Law service for a certain period. It is, indeed, regrettable to see from the report that unless more support is forthcoming this work of training must be curtailed. Subscriptions generally fell off in 1896, though it is satisfactory to see that those from guardians amounted to a little more than in the previous year. The due care of the sick in our workhouses should appeal with the greatest force to all charitable people, and to such we strongly recommend this excellent association. The visitor's report for the year—Miss Fynes-Clinton has visited 22 infirmaries during 1896—should be carefully studied, showing as it does the state of things still possible in too many workhouse wards. The committee may well suggest that "guardians will find in it some explanation of the frequent changes in their nursing staff," of which they so often complain. The association wants two things—more money to train probationers and more applications from suitable candidates. Vacancies for training have had to be relinquished because of this lack of suitable applicants—a difficulty now greatly increased (1) by the widespread reluctance to enter the Poor Law service while the new Superannuation Act remains as it is; and (2) by the ever-increasing impossibility of the position which trained nurses are required to accept in the smaller unions. Once the Local Government Board can be induced to issue the new Nursing Order, urged upon it by all who have expert knowledge on the subject, and the conditions of nursing at workhouses are made possible, there will be no lack of candidates, and it is certain that an association doing the work of the Workhouse Infirmary Nursing Association ought not, and surely will not, ask in vain for more financial support.

### Appointments.

### MATRONS.

Foyle Hill Fever Hospital, Londonderry.—Miss M. R. Daly has been elected Matron to the above hospital. She received three years' training in Sir P. Dun's Hospital, Dublin, and has been for the past four years on the private nursing staff of that hospital. We wish Miss Daly every success in her new sphere of duty.

East London Nursing Society.—Miss Mary Monkhouse has been appointed Matron of the Shadwell District of the East London Nursing Society. Miss Monkhouse was trained at Winchester Hospital, and for eighteen months held the post of charge nurse at Sheffield Infirmary. She is a Queen's nurse.

### Minor Appointments.

Ballymena Union.—Miss Rosanna Irvine has been appointed Nurse at this Union. She was trained at the Union Infirmary, Belfast, and was subsequently nurse at the Belfast Workhouse.

### The Johannesburg Hospital and the Nurses.

THE following extracts from a letter from Miss Young, Lady Superintendent of the Johannesburg Hospital, will be read with interest. Miss Young, it will be seen, confirms the view of the matter taken by Nurse "C. E. J." and Mr. H. S. Binney in their recent letters. Miss Young writes:—

"I brought out twenty-nine nurses with me in January, 1896, the Board paying first class expenses for each nurse from London to Johannesburg. On arriving at Capetown I was told we were to stay at the Grand Hotel from Friday to Monday, the Board paying all expenses, and, as many of the nurses had friends at the Cape, it was very nice for them. When we reached Johannesburg we found the nurses' new quarters were not ready for them, not through any fault of the Board's, but owing to the threatened war all the workmen had fled from Johannesburg and left the quarters half built. Dr. Van Niekerk, the resident surgeon, kindly gave his house up to us, we had meals there, and a few nurses with myself had rooms there also, but the majority of the nurses had to walk three-quarters of a mile night and morning, to a house hired by the Board, which was very hard on them after a heavy day's work, but as houses are most difficult to get it could not be avoided.

"When we had only been a fortnight here and had not got settled in the work, the terrible dynamite explosion took place, adding tenfold to our work, and as it is impossible to get help here as one can in England it made it a great strain on the nurses. It was impossible, with this extra pressure of work, to arrange days off for the first month, but, as we all had a month's rest before beginning work, it was not such a great privation as if the nurses had been hard at work previously. Since that time they have all had days off each month and two hours daily off duty, with half an hour for dinner and the same for tea, and whenever the work in any of the wards is slack I arrange for the nurses to get a longer time off duty, and I can safely say that no nurse has asked for extra time without my granting it.

"I should like to correct the statements made about the two nurses who died of typhoid. The work was very hard at the first, but, as neither of the two were strong, they had not stamina to pull through a serious illness, and they ran no more risk of getting typhoid than their fellow nurses (the marvel is how we all escape as we do). Nurse Chester was seven weeks in a private ward (the charge of which is 17s. 6d. a day to patients), and Nurse Ellen Smith, at her own request, was in a ladies' ward of six beds (the usual charge being 12s. a day). They had devoted attention and nursing from their fellow nurses both night and day, and, besides our three house doctors, they had several others in consultation, while whatever was ordered was given. When they died the Board paid all expenses of the funerals, and they, with the doctors and nurses, subscribed £45 and put up two crosses over their graves. Two other nurses (Butler and Jensen) also had typhoid; they were nursed in private wards (for which patients pay 17s. 6d. a day), and they had the same care and attention, and when they were able to go away the Board sent them to Durban at the expense of the hospital, which cost altogether £26 12s.

"The nurses' quarters are certainly not what could be desired, but considering the building cost £8,000, it shows that the board did not mean them to be badly housed. The rooms are divided into too small a portion for each nurse, and are separated with white curtains, but this is to be remedied, as the board are having a second storey built on, which will give twenty single rooms; the present rooms will then be divided into large cubicles for the probationers.

"I enclose a diet sheet; the food is better than in many English hospitals, one great thing being that the meals are served on hotel principles—each nurse gets her food quite hot and freshly served.

"If the nurses leave in a straightforward manner the Board are not hard upon them; for instance, one nurse left to become matron of a hospital out at one of the mines, and she paid expenses, £53 16s. 3d.; three others have left to be married, one had only been here about three months, she forfeited £53 16s. 3d.; another paid £50, and another £40, according to the time each had worked.

"The nurses go on duty at quarter-past seven and leave the ward at eight o'clock (except the charge nurses, who remain on to a quarter-past eight to give the report). The night nurses go on at ten minutes past eight and leave at half-past seven. There is a Kaffir boy on night duty in each ward, they attend to the lavatories and do all dirty work. At present my staff consists of twenty-two trained nurses and seventeen probationers; we nurse 176 patients. The Roman Catholic nuns nurse all the black men and a few white patients. Considering that we are in the Transvaal in a place only nine years old, I think it must be admitted that the nurses can hardly be accurately described as 'the unfortunate members of the profession now undergoing shabby treatment at the Johannesburg Hospital.'

"The Board are wishful to do all they can to make the nurses comfortable, but even in England it takes time to make improvements, and out here it is more difficult still. I have some very good and delightful nurses on my staff who are very angry to think their friends are unnecessarily worried about them owing to the untruthful accounts in the papers. I shall be very glad if publicity can be given to these facts.

"The nurses have just had a present of a very good new piano from a member of the Board; he has also given them free tram tickets, and all the members are wishful to make the nurses happy. The work is splendid, and I am having the probationers thoroughly trained; they have weekly lectures and I hope to turn out some really good useful nurses after their 3 years' training."

The following is a sample of the dietary, taken from one day's meals:—Breakfast, porridge, sausages; dinner, boiled mutton, vegetables, pastry; tea, bread and butter and jam; supper, mince, vermicelli pudding. Night nurses have the same as day nurses for dinner and supper; tea, bread and butter, eggs or sardines, and jam during the night; and for lunch, a glass of milk and bread and butter—on cold days a cup of cocoa is given instead.

### Everybody's Opinion.

[Correspondence on all subjects is invited, but we cannot in any way be responsible for the opinions expressed by our correspondents. No communication can be entertained if the name and address of the correspondent is not given, or unless one side of the paper only is written on.]

### WARD ETIQUETTE.

"A READER" writes: I would refrain from trespassing upon the space of your much valued paper did I not feel keenly on a subject in respect to which from experience in various hospitals in the country I know that much good might be done if my suggestions were taken to heart. I refer to the unfortunate practice of treating nurses to an unnecessary amount of language in the wards and under the very ears of the patients. We are candid to admit that mistakes are made by the probationer, or the assistant nurse, or even the ward sister in carrying out their many and intricate duties, but surely they are not such as to justify the exposure of the shortcomings of those who in their turn are responsible for exerting their authority over the patients themselves. I do not wish for one moment to defend the nurse who is almost habitually given to carelessness, and I know that at times there are defects which must be pointed out in the wards, but these are not the cases to which I refer. I have seen these cases, but oftener has my blood boiled when for some trivial offence I have heard the matron's voice ringing through the ward, and not in over complimentary terms reminding the nurse of the proper way the work should be done. It is not always that the correction is justifiable, but even when it is is it not far better to leave the "rowing" to some other place and not to show up the probably over anxious nurse before her patients. Probably there are some of your readers who may differ from my view, but I feel that there are many whose sympathies will run with mine, and I can only hope that some one may take to heart my humble suggestion.

### Death in our Ranks.

MISS ALICE MARY WALLICK, Queen's Nurse, late superintendent of the Woolwich Branch of Queen Victoria's Jubilee Institute, and newly appointed inspector, died on February 26th at Sibton, Suffolk, of typhoid fever. She is deeply regretted by all who were privileged to know her, and in her the Queen's Institute has lost a most loyal and devoted worker.

### For Reading to the Sick.

LENT.

**Verses.**

THE solemn season calls us now
A holy fast to keep,
And see within the temple how
Both priests and people weep.
But come not thou with tears alone,
Or outward form of prayer,
But let it in thy heart be known
That penitence is there. —*J. Chandler.*

Humbly, now, with deep contrition,
We Thy mercy, Lord, entreat,
Now, as mourning, weeping, kneeling,
We bow down before Thy Feet.
Father, in the day of anguish,
And of darkness and of shame,
Cling we to that precious promise,
Made to us in Jesu's Name.
—*A. Cambridge.*

Christian ! dost thou feel them,
How they work within,
Striving, tempting, luring,
Goading into sin ?
Christian ! never tremble,
Never be downcast;
Smite them by the virtue
Of the Lenten fast.
Christian ! dost thou hear them,
How they speak thee fair:
"Always fast and vigil;
Always watch and prayer ? "
Christian, answer boldly,
"While I breathe, I pray";
Peace shall follow battle,
Night shall end in day.
—*S. Andrew of Crete.*

**Reading.**

Turn ye even to Me, saith the Lord, with all your heart, and with fasting, and with weeping, and with mourning; and rend your heart, and not your garments, and turn unto the Lord your God.—*Joel* ii. 12, 13.

Lent is the solemn time which the Church tells us to keep before Easter. The meaning of the word is "spring," because it comes in the spring. At different times its length has differed, but it lasts now for forty days only. These days are what are called fast days. Fasting means going without food. It is not to be supposed that it pleases God, our heavenly Father, to see His children made uncomfortable. He likes us to be happy and enjoy ourselves in an innocent way, and if we were free from sin and living in Paradise, we should not have to fast. . . It is for this reason that we fast: "To keep under our bodies, and bring them into subjection." Otherwise we may become unable to serve God or to enjoy real happiness.—*L. R.*

The true idea of mortification is that it is the love of Jesus, urged into that shape partly in imitation of Him, partly to express its own vehemence, and partly to secure its own perseverance. There can be no true or enduring love without it, for a certain amount is requisite in order to avoid sin and keep the commandments.

The above words apply, perhaps, more directly to one class of persons than another. There are many among us who are sick and ill, whose physical condition therefore precludes them from any sacrifice in this direction. But there are other sacrifices which are of equal value, which it is in the power of all of us to offer up to the Great High One who set this world of ours an example of sacrifice throughout His earthly career. Little acts of kindness, offered in His name, little words of love and sympathy to His flock here—these, equally with other works entered into in the Holy Spirit of the season, will be helpful to us and those around us; and, as such, will be understood by God, and acceptable in His sight.—*A. M. P.*

### Notes and Queries.

*Nursing in Australia.*

(160) Would a trained nurse with three years' certificate be likely to find work in Sydney, New South Wales, or the neighbourhood, in hospital or otherwise? To whom should she apply?—*Daisy.*

We do not think there is any demand for English nurses in Australia, where for years past systematic training has been given in the hospitals, and numbers of well-trained nurses are available. You might write to Miss McGahey, matron of the Prince Alfred Hospital, Sydney, on the subject.

*Advice Wanted.*

(161) Can you tell me of a hospital or home to which a month-old baby with hare-lip could be received? Its mother would pay something for its nursing until it is able to undergo an operation; meantime she cannot attend to it properly, having business to attend to.—*R. O'R., Aldershot.*

Do you mean that the child is already under medical advice, and only awaiting treatment, or that you want it to be taken into a hospital where the operation would be undertaken? There is a cottage hospital at Farnham, or why not take the child to the County Hospital at Guildford? These institutions are within easy reach. A little further off would be the Redhill and Reigate Cottage Hospital. Try at one or other of these institutions.

*A Question of Etiquette.*

(162) A lady nurse asks if it be the correct thing, when nursing a private case, to address the doctor as "Sir," and to give the same title to patients or their friends.—*Dubitans.*

Perhaps some of our readers will give their views upon the matter for the benefit of our correspondent. In the case of the doctor we certainly think the hospital form of address may well be adhered to. The nurse is under the direction of the medical man, and the term comes as naturally from her under the circumstances as it does from a subaltern to his colonel.

*Dictionary.*

(163) I am studying for the L.O.S. examination, and want to obtain a second-hand dictionary of midwifery. Can you recommend me what to get, and tell me of a second-hand bookseller?—*Midwife.*

Any good dictionary of medical terms will answer your purpose—Hoblyn's or Douane's—and you could probably get them second-hand. Write to Mr. Brown, bookseller, Bishop's Road, W., for a catalogue. "The Nurse's Dictionary" (Scientific Press), 1s., would probably meet your requirements.

*Specimens for Hire.*

(164) Can you tell me where I could hire a pelvis, skull, &c., as specimens? Also please tell me where appliances are made for deformed feet to wear as a boot?—*Nurse A.*

Surgical instrument makers often supply specimens to medical students. They are, however, rather expensive. As regards your second question, such appliances are made by surgical instrument makers, but nothing of the kind should be obtained and worn without proper medical advice.

*Death Certificates.*

(165) A "Midwife Holding a Certificate" asks if she can give a certificate of death for a newly-born infant, should it live a few hours.

Certainly not; certificates of death can only be given by a qualified medical man.

*"Cottage Nursing."*

(166) Can I get any training for a young woman fond of nursing, enough to enable her to assist a village nurse in general nursing, without her going through a regular hospital training, for which she is not strong enough?—*Rector's Wife, Dorset.*

Short periods of training, which might suit a person wishing only to assist a village nurse, are given by the Dorset County Nursing Association. Its headquarters are at Blandford.

*Lady Dufferin's Fund.*

(167) Please give me the address of this Fund for sending medical women and nurses to India?—*Matron.*

Write to the assistant secretary, Miss Edith Heather-Bigg, 14, Radnor Place, Hyde Park, London, W.

*Nursing in India or South Africa.*

(168) What is the best way to set about obtaining work in hospitals abroad, either in India or South Africa?—*C. H.*

Watch our advertisement columns, write direct to the matrons of hospitals in that part of the world to which you wish to go (you will find a list in Burdett's "Hospitals and Charities," Scientific Press, 28, Southampton Street), and apply to the Colonial Nursing Association, Imperial Institute, London, S.W. In the future all Colonies requiring English nurses are certain to obtain them through the medium of this Association.

*Disinfection, &c.*

(169) I am nursing a case of typhoid fever. Where in the North of London and for how much can one have a mattress disinfected? (2) Has the L.O.S. a badge for those who have passed its examination? (3) Would it be possible to start a post-card examination for nurses who have finished their training and want to be kept up to the mark?

Communicate with the Medical Officer of Health of the district in which the case may be situated. 2 Not that we are aware of.

*Enquirer,* who asks about a home for poor gentlemen, and "*K,*" who wants information respecting homes for incurables, are reminded that name as well as address must accompany all queries requiring an answer in this column.

*Home Wanted.*

(170) Can you tell me of a home in or near London where an old gentleman of 71 could be taken in? He needs a little nursing, and could pay 10s. to 15s. a week.—*Old Age.*

We have made inquiries and fear it will be difficult to find such a home. Look down the list of homes in Burdett's "Hospitals and Charities," and see if any there mentioned fit the needs of the case. Most institutions for the special reception of aged people are only for the very poor or else are strictly local, as in the case of almshouses. Possibly some of those mentioned in the above book may suit the circumstances of this case, of which we cannot judge without further particulars.

